# Multiparametric bone MRI can improve CT-guided bone biopsy target selection in cancer patients and increase diagnostic yield and feasibility of next-generation tumour sequencing

**DOI:** 10.1007/s00330-022-08536-6

**Published:** 2022-01-29

**Authors:** Ricardo Donners, Ines Figueiredo, Nina Tunariu, Matthew Blackledge, Dow-Mu Koh, Maria de los Dolores Fenor de la Maza, Khobe Chandran, Johann S. de Bono, Nicos Fotiadis

**Affiliations:** 1 Department of Radiology, Royal Marsden Hospital, Downs Road, Sutton, SM2 5PT UK; 2 Department of Radiology, University Hospital Basel, Petersgraben 4, 4031 Basel, Switzerland; 3 The Institute of Cancer Research, 15 Cotswold Road, Sutton, SM2 5NG UK; 4 Cancer Research UK Cancer Imaging Centre, The Institute of Cancer Research, 15 Cotswold Road, Sutton, SM2 5NG UK; 5 The Royal Marsden NHS Foundation Trust, Downs Road, Sutton, SM2 5PT UK; 6 Department of Interventional Radiology, Royal Marsden Hospital, 203 Fulham Rd, London, SW3 6JJ UK

**Keywords:** Neoplasms, Image-guided biopsy, Magnetic resonance imaging, Genomics, Bone marrow

## Abstract

**Objectives:**

To evaluate whether multiparametric bone MRI (mpBMRI) utilising a combination of DWI signal, ADC and relative fat-fraction (rFF) can identify bone metastases, which provide high diagnostic biopsy yield and next-generation genomic sequencing (NGS) feasibility.

**Methods:**

A total of 150 CT-guided bone biopsies performed by interventional radiologists (3/2013 to 2/2021) at our centre were reviewed. In 43 patients, contemporaneous DWI and rFF images, calculated from 2-point T1w Dixon MRI, were available. For each biopsied lesion, a region of interest (ROI) was delineated on ADC and rFF images and the following MRI parameters were recorded: visual classification of DWI signal intensity (SI), mean, median, 10th and 90th centile ADC and rFF values. Non-parametric tests were used to compare values between tumour positive/negative biopsies and feasible/non-feasible NGS, with *p*-values < 0.05 deemed significant.

**Results:**

The mpBMRI combination high DWI signal, mean ADC < 1100 µm^2^/s and mean rFF < 20% identified tumour-positive biopsies with 82% sensitivity, 80% specificity, a positive predictive value (PPV) of 93% (*p* = 0.001) and NGS feasibility with 91% sensitivity, 78% specificity and 91% PPV (*p* < 0.001). The single MRI parameters DWI signal, ADC and rFF failed to distinguish between tumour-positive and tumour-negative biopsies (each *p* > 0.082). In NGS feasible biopsies, mean and 90th centile rFF were significantly smaller (each *p* < 0.041). Single ADC parameters did not show significant difference regarding NGS feasibility (each *p* > 0.292).

**Conclusions:**

MpBMRI utilising the combination of DWI signal, ADC and rFF can identify active bone metastases, which provide biopsy tissue with high diagnostic yield and NGS feasibility.

**Key Points:**

*• Multiparametric bone MRI with diffusion-weighted and relative fat-fraction images helps to identify active bone metastases suitable for CT-guided biopsy.*

*• Target lesions for CT-guided bone biopsies in cancer patients can be chosen with greater confidence.*

*• CT-guided bone biopsy success rates, especially yielding sufficient viable tissue for advanced molecular tissue analyses, can be improved.*

## Introduction

The role and frequency of CT-guided metastatic bone biopsies is increasing to determine prognosis, predict response and detect treatment resistance in cancer patients [1, 2]. However, bone metastases, especially when sclerotic, have been notoriously challenging to biopsy, because the amount of obtained viable tissue is scant, requires decalcification and is often inadequate for molecular analyses. Published success rates of CT-guided bone biopsies for tumour diagnosis range between 58 and 90% [1, 3–12]. For more complex analysis, such as next-generation genomic sequencing (NGS), lower success rates between 39 and 82% were reported [1, 6, 10, 13].

A major contributor to the success or failure of CT-guided bone biopsies is the accurate identification of active/viable metastases versus scarring / non-viable areas and normal bone marrow. Conventional CT is unspecific and cannot inform on bone tumour viability. Reported CT features favouring a successful bone biopsy include targeting CT-lucent lesions, the periphery of high CT attenuation lesions and progressive lesions [6, 14]. To compensate for the shortcomings of CT, some authors advocate using PET/CT to identify bone biopsy targets [15, 16]. Recently published data on using prostate-specific membrane antigen (PSMA)-PET/CT for bone biopsy targeting in prostate cancer patients improved NGS feasibility rates up to 70–90% [12, 17, 18]. Remarkably, although being recognised as the gold standard imaging method for detecting malignant bone diseases, MRI features that are associated with a positive bone biopsy or NGS outcome have not been described [19, 20].

Mature, yellow bone marrow MRI signal is mostly generated by fat- and to a small degree water-based protons. In red, haematopoietic marrow, water-based protons contribute more to the overall signal. In contrast, viable, cellular bone metastases replace bone marrow and MRI signal is almost exclusively generated by water-based protons. Response to therapy results in metastatic tumour cell kill and, in some cases, return of normal, fatty bone marrow. These differences in biochemical composition of bone marrow and cellularity of viable and treated metastases can be exploited to generate contrast with MRI.

DWI is a functional imaging technique, acquired by applying diffusion sensitising gradients (described by the *b*-value) to a fat-saturated, T2-weighted, hence fluid-sensitive sequence, which visualises differences in water mobility. Cellular malignancy generates high signal on heavily diffusion-weighted images (high *b*-values), while surrounding normal tissue signal is suppressed. The degree of water mobility is quantified by the apparent diffusion coefficient (ADC), derived from monoexponential fitting of the signal decay observed between lesser (low *b*-value) and higher (high *b*-value) diffusion weightings. ADC is recognised as a quantitative biomarker inversely correlating with tumour and bone marrow cellularity [21, 22], which can be used to avoid misinterpretations of DWI signal, due to T2-shine-through high DWI signal caused by long T2 decay as encountered in necrotic metastases responding to therapy, rather than true diffusion restriction generating high ADC. However, the unique trabecular bone matrix and variable fat/water ratio may cause high DWI signal and low ADC of healthy marrow. Dixon MRI-derived relative fat fraction (rFF) images can complement DWI, by visualising and quantifying voxel fat content [23, 24]. Higher rFF percentages are found in healthy bone marrow or treated metastases, while active lesions have very low rFF [25]. Hence, the combination of DWI and rFF allows for identification of viable bone metastases.

The purpose of this study was to evaluate whether multiparametric bone MRI (mpBMRI) utilising a combination of DWI signal, ADC and rFF can identify active bone metastases, which provide high diagnostic biopsy yield and NGS feasibility.

### Materials and methods

This retrospective single-centre study was conducted according to the legal regulations of the local research and ethics committee of our institute. Requirement for informed patient consent was waived.

**Fig. 1:**
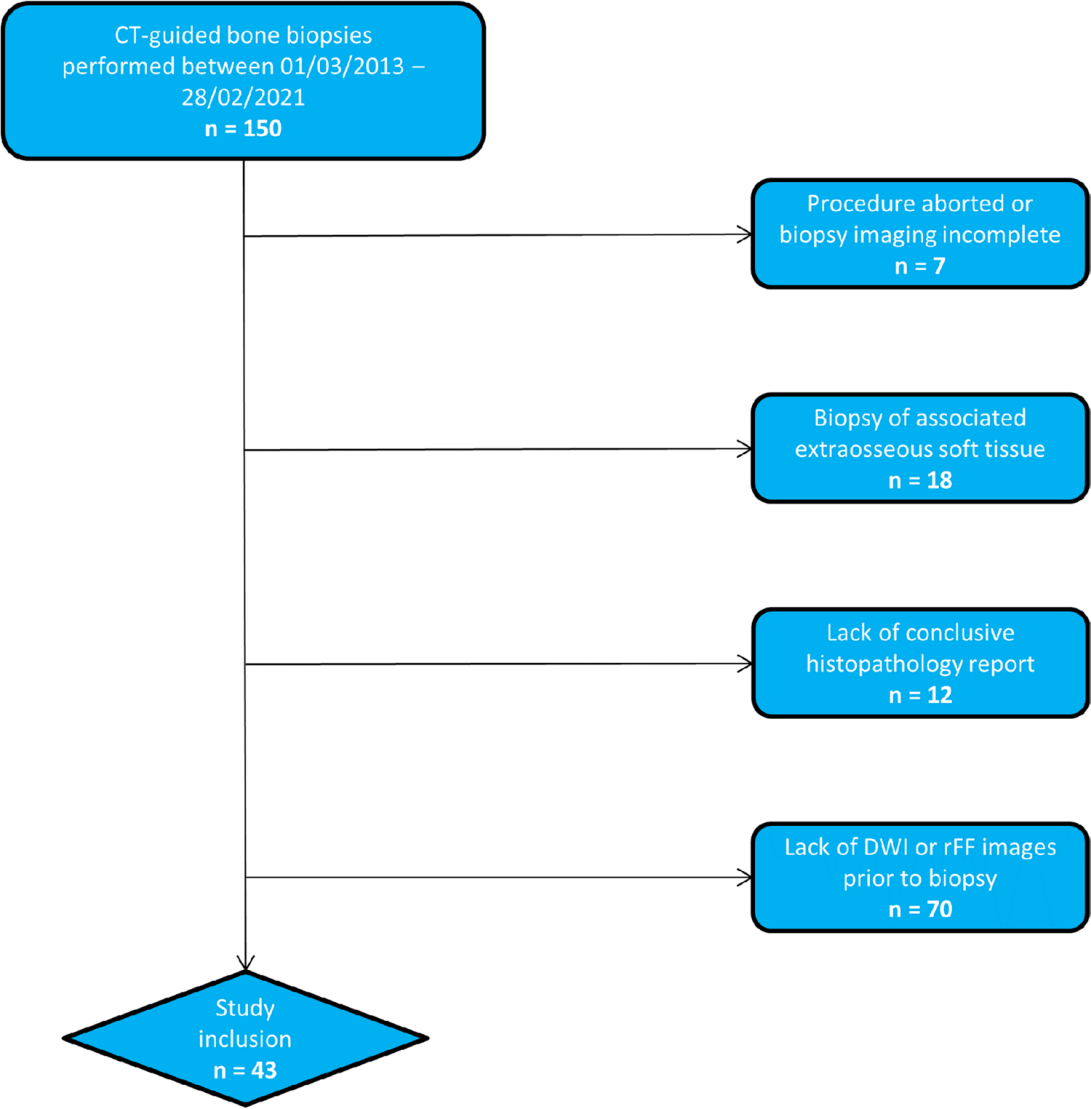
Study inclusion process, n, number of patients; rFF, relative fat fraction MRI

Study inclusion criteria were as follows: CT-guided bone biopsy performed in our department between 01/03/2013 and 28/02/2021, availability of the biopsy CT (Bx-CT) allowing for unequivocal identification of the biopsied lesion, availability of the conclusive histopathology result and contemporaneous MRI including DWI, ADC and rFF images performed within 3 months prior to the biopsy. Within the institute’s radiology information system, data of 150 CT-guided bone biopsies performed in the given time interval were identified. Figure 1 visualises the patient recruitment process. In total, 43 CT-guided bone biopsies performed in five female and 38 male patients, mean age 66 ± 7 years, were included. Primary malignancies in declining order were: 36 prostate adenocarcinoma, four breast adenocarcinoma, one B cell lymphoma, one pulmonary squamous cell cancer and one clear renal cell cancer.

#### CT-guided bone biopsy procedure

A specialised interventional radiologist, experienced in CT-guided bone biopsies or a supervised interventional radiology fellow performed the biopsies included in this study. All procedures were performed under local anaesthesia and conscious sedation. Target lesions were selected based on composite assessment of all available imaging for each patient at the time of biopsy. Jamshidi™ (Becton, Dickinson and Company) and Madison™ (Merit Medical) 11–13G biopsy needles were used. On average three core samples were obtained per biopsy.

##### Histopathology

Histopathology results were available in the electronic patient records of our hospital. After the biopsy cores were obtained, samples were fixed for 24–30 h and decalcified in an EDTA solution for 2 days to preserve the DNA quality for NGS. Tumour content was assessed by a trained pathologist. Bone biopsy samples were deemed suitable for NGS when showing at least 150 cancer cells on H&E-stained, 2-μm-thick specimen slices. 10 × 6-μm sections were prepared for sequencing. Whenever possible, a minimum of 20% tumour content was pursued by either coring or macro dissection following Nuclear Fast Red (NFR) staining in cases where the tumour cells were not dispersed throughout the tissue sample. Biopsy specimen containing less than 150 tumour cells on H&E stained slices were deemed not suitable for NGS by the pathologist and consequently unsuccessful in the context of genomic analysis.

##### Imaging

**Fig. 2:**
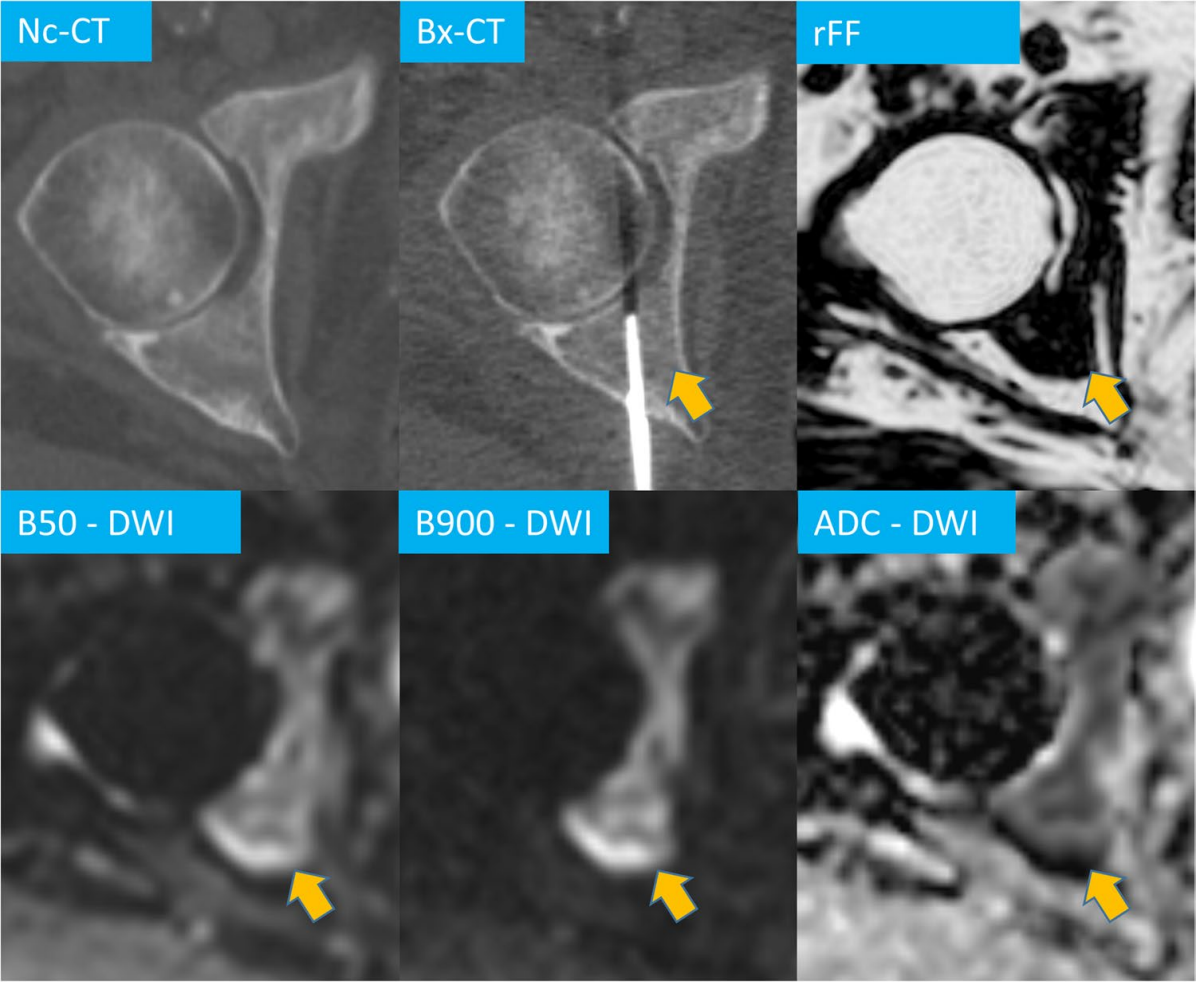
Multiparametric MRI of a right acetabulum metastasis in a 60-year-old prostate cancer patient. Relative fat fraction (rFF), diffusion-weighted (DWI) b50 and b900 images and apparent diffusion coefficient (ADC), biopsy CT (Bx-CT). Note how the lesion cannot be identified on the CT images

Free-breathing DWI and breath-hold T1-weighted 2-point volume interpolated breath-hold examination (VIBE) Dixon parameters as performed on the institute’s 1.5 T MRI scanner (MAGNETOM Avanto, Siemens Healthineers) are summarised in Table 1. From the VIBE Dixon fat-only (FO) and water-only (WO) images, relative fat fraction (rFF) images were calculated as rFF = $\frac{FO}{FO+WO}$. Exemplary images as acquired by the described protocol showing acetabular metastases with corresponding CT images are shown in Fig. 2.

**Table 1:** MRI parameters

Parameter	DWI	T1 VIBE Dixon
Plane	Axial	Axial
Slice thickness (mm)	5	5
*b*-values	50, 900	
Field of view (mm)	400 × 390	400 × 390
Acquisition matrix	150 × 144	256 × 180
Repetition time (ms)	14,600	13.9
Echo time (ms)	65	2.39
Number of averages	4	1
Flip angle	90°	10°
Bandwidth (Hz/pixel)	1961	470
Acquisition time (min:s)	2:21	0:33

*DWI* diffusion-weighted imaging, *VIBE* volumetric interpolated breath-hold examination

#### Image evaluation

Imaging analysis was performed by a diagnostic and interventional radiology fellow, with three years of experience in dedicated MRI of malignant bone disease, trained in performing CT-guided bone biopsies. Images were evaluated on commercially available post-processing software (OsiriX, version 56, PixmeoSARL). MpBMRI was used as single identifier to distinguish between successful and unsuccessful biopsies in the diagnostic and genomic analysis context, irrespective of the availability or features on other imaging modalities such as PET/CT or CT.

**Fig. 3:**
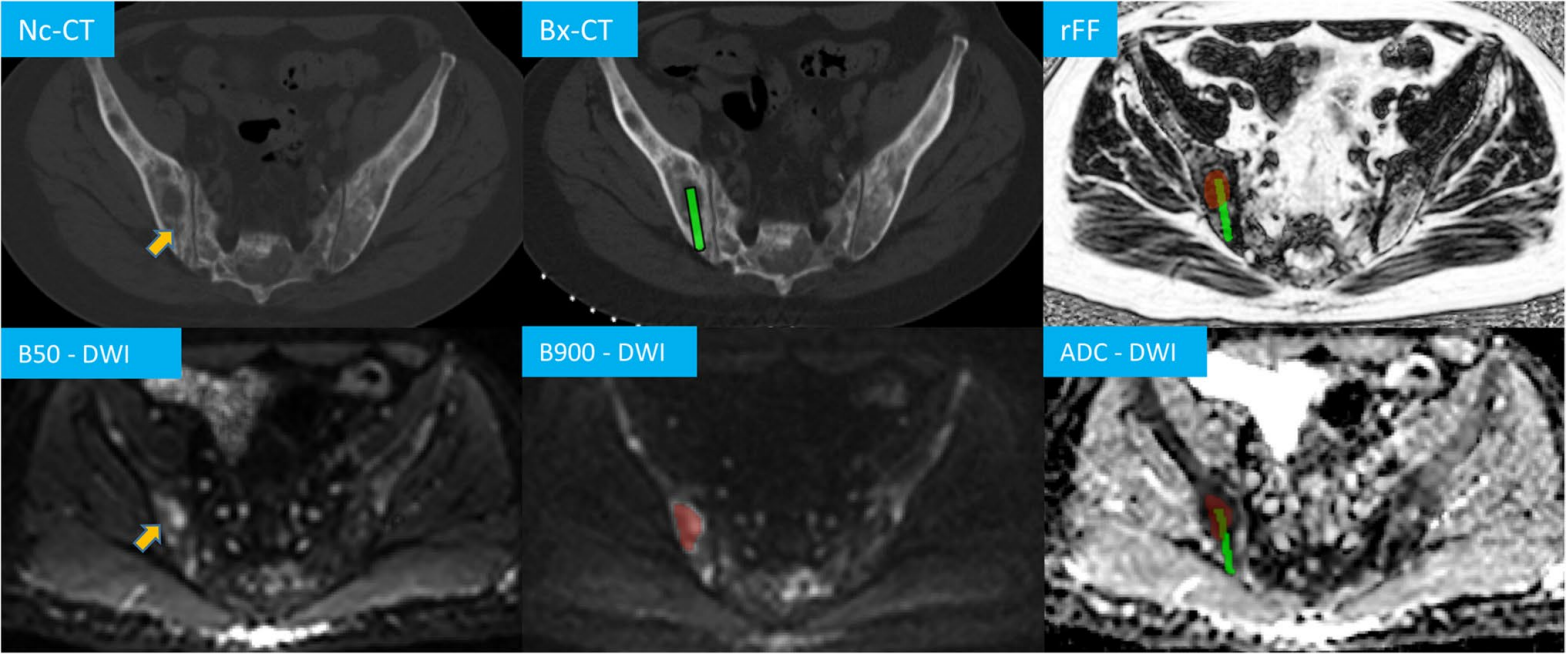
CT and multiparametric MRI of a right iliac bone metastasis in a 53-year-old prostate cancer patient. The biopsy tract (upper orange arrow) on the non-contrast (Nc) CT performed after the biopsy was delineated (green) and transferred onto the planning biopsy CT (Bx-CT), relative fat fraction (rFF), diffusion-weighted (DWI) b900 and ADC images. It intersects the delineated metastasis (light red, orange arrow on b50 image)

First, on the Bx-CT images, the biopsy site was identified. CT appearance and CT attenuation characteristics were not analysed in this study. Using OsiriX, DWI and rFF images were matched to the corresponding imaging slice. The DWI signal intensity (SI) of the biopsied lesion on b50 and b900 was documented. High SI was noted when the lesion’s signal was not-suppressed. An ROI, encompassing the high DWI b900 SI lesion was generated and copied onto the ADC map. The same lesion was delineated on rFF images. ADC and rFF mean, median and 10th and 90th centile values were derived. The biopsy tract was delineated on Bx-CT images and copied onto the corresponding DWI, ADC and rFF images. This was achieved by using an in-house developed, python™ based registration tool applied within OsiriX that required the definition of reference points on anatomical landmarks surrounding the tract on Bx-CT and MR images. This allowed the software to transfer the ROIs between different modalities and adjust it to different resolutions Fig. 3. When the biopsy tract ROI did not intersect the lesion ROI or no lesion could be identified on DWI and rFF images at the biopsy tract level, biopsies were termed as “procedural miss defined by mpBMRI”.

#### Statistical analyses

Statistical analyses were performed using commercially available software (IBM SPSS Statistics Version 25, IBM Corp.). Qualitative, nominal scaled variables were compared using Fisher’s exact test. Quantitative variables were compared using Mann–Whitney-U tests. A *p*-value < 0.05 was deemed statistically significant. In cases of significance of continuous variables, ROC curve analyses were performed and optimised threshold values were extracted using Youden’s index to distinguish between tumour-positive and tumour-negative biopsies and NGS feasible versus NGS non-feasible biopsies. Multiparametric serial testing, combining DWI signal characteristics, ADC and rFF in an “and-configuration” was performed to distinguish between successful and unsuccessful biopsies and the combined Youden Index allowed for selection of optimised, combined threshold values for the quantitative parameters ADC and rFF.

## Results

33/43 (76%) biopsies were tumour-positive and NGS was feasible in 22/31 (71%) evaluated samples. The average time interval between biopsy and MRI was 32 ± 20 days. 8/10 tumour-negative biopsies contained insufficient tissue for a final diagnosis, one sample was necrotic and one contained normal bone and scar tissue. No procedural misses were identified; hence, all biopsies were included for further analysis.

## DWI signal

High b50 and b900 DWI signal was displayed by all 33 tumour-positive biopsies and 7/10 (70%) tumour-negative biopsies (*p* = 0.01). High DWI signal had 100% (95% CI 89–100%) sensitivity and 30% (95% CI 7–65%) specificity and a positive predictive value (PPV) of 83% (95% CI 76–88%) for identification of tumour-positive biopsies. All 22 biopsies that were feasible for NGS displayed high DWI signal. 7/9 (78%) of biopsies not NGS-feasible also showed high DWI signal (*p* = 0.077).

## ADC

The average lesion ADC ROI volume was 1.92 ml. Table 2 shows and compares the derived histogram parameters of the biopsied lesions. Although ADC values in tumour-positive biopsies and in cases of NGS feasibility were generally lower compared with tumour-negative and NGS non-feasible biopsies, due to the large standard deviations there was significant overlap and no statistical significance (each *p* > 0.121). The mean ADC range of biopsies feasible for NGS was 332–1098 µm^2^/s and for NGS non-feasible biopsies 750–1399 µm^2^/s.

**Table 2:** Apparent diffusion coefficient measurements

	Biopsy diagnosis					Genomic sequencing				
	Positive (33)		Negative (10)			Feasible (22)		Not feasible (9)		
	Mean	SD	Mean	SD	*p*-value	Mean	SD	Mean	SD	*p*-value
Mean ADC	829.4	201.7	996.9	1348.7	0.313	805.9	184.5	958.9	276.4	0.292
Median ADC	816.1	200.1	988.0	372.2	0.371	796.3	180.9	931.4	273.8	0.356
10th centile ADC	643.9	156.0	776.8	281.1	0.386	620.4	146.7	712.7	203.8	0.428
ADC	1038.5	300.3	1216.5	416.2	0.419	1001.3	267.4	1215.3	373.1	0.273

*ADC* apparent diffusion coefficient in µm^2^/s, *SD* standard deviation

## rFF

The average lesion ROI volume was 2.15 ml for rFF. Table 3 summarises the derived rFF parameters. Although mean, median and 90th centile rFF were smaller in tumour-positive versus tumour-negative biopsies, findings were non-significant (each *p* > 0.082). Regarding NGS feasibility, significantly smaller mean and 90th centile rFF percentages were derived for NGS feasible biopsies (*p* < 0.041).

**Table 3:** Relative fat fraction measurements

	Biopsy diagnosis					Genomic sequencing				
	Positive (33)		Negative (10)			Feasible (22)		Not feasible (9)		
	Mean	SD	Mean	SD	*p*-value	Mean	SD	Mean	SD	*p*-value
Mean rFF	14.0	63	17.3	7.2 I	0.082	14.5	5.6	19.5	6.0	0.041
Median rFF	13.2	6.1	16.1	6.8	0.133	13.8	5.3	18.2	6.0	0.086
10th centile rFF	7.5	6.9	7.2	3.0	0.419	6.2	2.5	8.4	3.3	0.147
90th centile rFF	23.1	10.2	28.3	12.1	0.105	23.9	9.6	31.7	9.3	0.026

*rFF* relative fat fraction in %, *SD* standard deviation

**Fig. 4:**
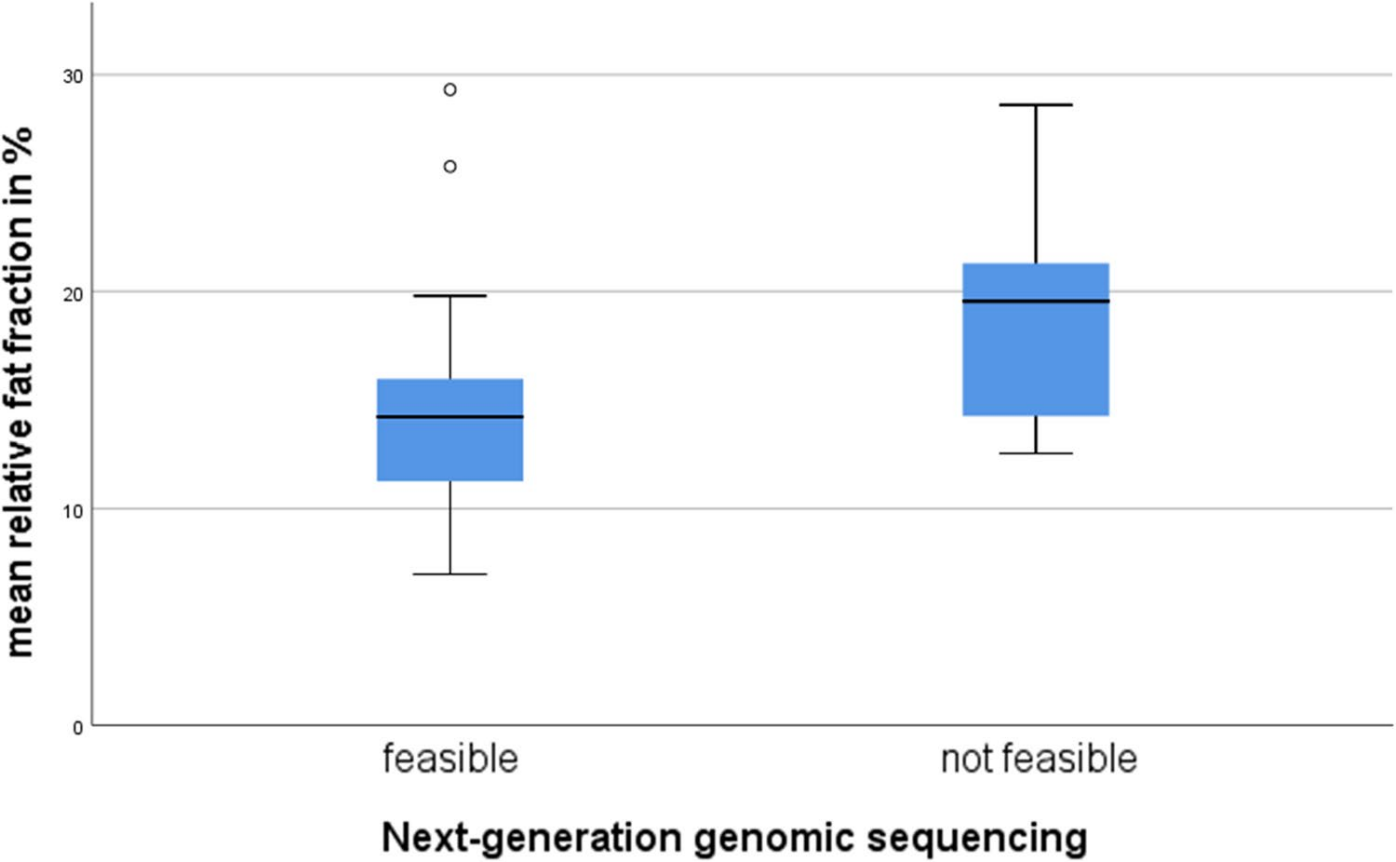
Boxplot visualising the discriminatory ability of mean relative fat fraction between biopsies feasible and not-feasible for next-generation genomic sequencing. Note how but two outlier genomic-sequencing feasible lesions lie below 20% relative fat-fraction

Mean rFF ROC analysis revealed an AUC of 0.74 (95% CI 0.55–0.93), the largest Youden Index at a 16.3% threshold, which identified NGS feasible biopsies with 77% (95% CI 55–92%) sensitivity, 67% (95% CI 30–93%) specificity, a PPV of 85% (95% CI 69–94%) and a NPV of 55% (95% CI 33–75%). In bone metastases providing samples feasible for NGS mean rFF ranged from 7–29% and when NGS was not feasible from 13–29%. As shown in the boxplot in Fig. 4, two outliers feasible for NGS had rFFs higher than 20%, while the other 20 lesions (91%) showed mean rFF lower than 20%.

For 90th centile rFF, ROC analysis revealed an AUC of 0.76 (95% CI 0.58–0.93) as discriminator for NGS feasibility. A 27.5% threshold showed the highest Youden Index, identifying NGS feasible lesions with 77% (95% CI 55–92%) sensitivity, 67% (95% CI 30–93%) specificity, a PPV of 85% (95% CI 69–94%) and a NPV of 55% (95% CI 33–75%).

## Multiparametric bone MRI discrimination with serial testing

**Fig. 5:**
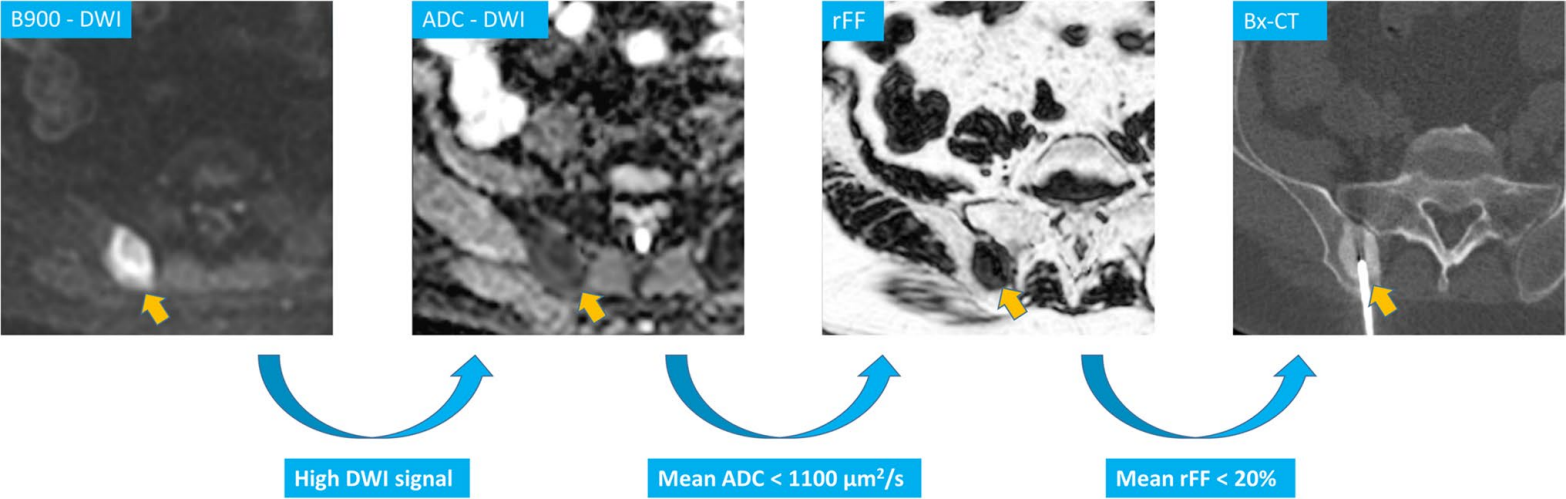
Right iliac crest metastasis in a 56-year-old prostate cancer patient and multiparametric MRI bone biopsy target selection algorithm, high signal DWI b900 diffusion-weighted images (DWI), low apparent diffusion coefficient (ADC), relative fat fraction (rFF), and consecutive biopsy CT (Bx-CT). Note that despite the sclerotic CT appearance, the biopsy samples were suitable for next-generation genomic sequencing

The parameter combination of high b50 and b900 DWI signal, a mean ADC threshold of 1100 µm^2^/s and a mean rFF threshold of 20% was identified for optimal target lesion selection. This simple algorithm employed in serial testing identified tumour-positive biopsies with 82% sensitivity, 80% specificity and 93% PPV(*p* = 0.001). The same 3-step approach identified NGS feasibility with 91% sensitivity, 78% specificity, and 91% PPV (*p* < 0.001). Table 4 summarises the performance of serial mpBMRI parameter testing using the described thresholds. The lesion selection process is visualised as a simple diagram in Fig. 5.

**Table 4:** Multiparametric bone MRI (mpBMRI) serial parameter testing performance for identification of successful biopsies

mpBMRI performance	Identification of tumour-positive biopsies	Identification of NGS feasibility
True positive	27/33	20/22
True negative	8/10	7/9
False positive	2	2
False negative	6	2
Sensitivity	82% (65–93%)	91% (71–99%)
Specificity	80% (44–97%)	78% (40–97%)
PPV	93% (80–98%)	91% (75–97%)
NPV	57% (37–74%)	78% (47–93%)

95% confidence interval in brackets; *NGS* next-generation genomic sequencing, *PPV* positive predictive value, *NPV* negative predictive value

## Discussion

This study showed that mpBMRI including DWI and rFF facilitates lesion selection to improve diagnostic tumour yield and NGS feasibility rates of CT-guided bone biopsies in cancer patients. The parameter combination high DWI signal, ADC < 1100 µm^2^/s and rFF < 20% could achieve 93% and 91% PPV for identification of tumour-positive and NGS-feasible biopsies, respectively.

The overall 76% success rate of CT-guided bone biopsies for diagnosis of malignancy and 71% feasibility rate of NGS of the reported cohort are within the ranges of the cited literature at 58–90% and 39–82%, respectively [1, 3–12, 17].

We recommend using the combination of DWI, ADC and rFF using the following approach: The first step should identify bone marrow, which fails to suppress on DWI, showing high SI on low and high *b*-value images. Omitting the possibility of imaging artefacts at this point, high DWI signal indicates the presence of a significant amount of water-based protons in what should physiologically be a fatty environment. Accordingly, all tumour-positive and NGS-feasible biopsy targets displayed high DWI signal. Our findings suggest that low DWI SI bone areas should be avoided for biopsy. Secondly, bone areas with high DWI signal should be further scrutinised on the corresponding ADC map. Based on our results, lesions displaying a mean ADC > 1100 µm^2^/s are not optimal biopsy targets. Lesion cellularity may be insufficient for successful NGS. Thirdly, lesions with ADC values < 1100 µm^2^/s should be confirmed on rFF images as areas without fatty bone marrow. In our study cohort, bone with a mean rFF < 20% yielded superior biopsy results compared with bone lesions displaying > 20% mean rFF. Consequently, targeting lower rFF bone lesions is advocated. Higher rFF percentages can indicate treatment response with returned marrow fat in a cancer patient and can result in biopsies with low active tumour content or tumour-negative biopsies.

The suggested three-step approach for identification of bone biopsy targets is simple, intuitive and can be used with standard oncological imaging protocols for malignant bone disease [19, 20]. DWI and Dixon sequences are provided on most MRI scanners. In addition to the excellent performance for selection of suitable bone biopsy targets, further benefits of using MRI as the target imaging modality are the higher spatial resolution of DWI and rFF in comparison to PET and lack of the blooming artefact often encountered with PET tracers. Furthermore, mpBMRI is neither tracer nor tumour specific and can be employed in a wide spectrum of cancer patients, also omitting the need for tracer application or ionising radiation. Moreover, dedicated DWI and rFF protocols can be acquired in less than 10 min and MRI has become widely available in most places.

As with all biomarkers, good repeatability and reproducibility are desirable to facilitate clinical implementation. Previous studies showed good ADC reproducibility of malignant bone lesions and normal bone with repeatability coefficients below 15% [26–29]. Two-point Dixon-derived rFFs are commonly employed in musculoskeletal imaging, have shown good correlation with MR spectroscopy [30], excellent inter-observer agreement for bone metastases [31] and mean rFF coefficients of variation below 12% in malignant bone lesions [28]. Nevertheless the suggested ADC and rFF values and their combination as utilised in the CT-guided bone biopsy target selection process need to be verified in a prospective setting.

This study has limitations. First, of the 150 CT-guided bone biopsy patients reviewed only 43 could be included. This was mainly for lack of mpBMRI including DWI and rFF. Second, the retrospective design creates inclusion bias. The bone lesions analysed in this study had already undergone a selection process to be chosen for tissue sampling. Third, this retrospective study cannot address a major issue in biopsy planning: while mpBMRI can improve biopsy target selection, ultimately the procedure will be performed CT-guided. Real-time fusion of CT and MR images could potentially improve biopsy results. Finally, MRI and especially DWI may be subject to a number of imaging artefacts affecting signal intensities and ADC calculation [32]. This highlights the need for an additional imaging parameter found in rFF and emphasises why parameters should be applied in a multi-step approach. We believe the chosen imaging criteria to be robust and applicable in a prospective setting, which is undergoing in our institution.

In conclusion, mpBMRI utilising the combination of DWI signal, ADC and rFF can identify active bone metastases suitable for biopsy, which provide high diagnostic yield and NGS feasibility. It should be considered part of the interventional imaging workup for biopsy lesion selection in cancer patients with bone metastases.

## Abbreviations

Bx-CT: Biopsy CT
FO: Fat-only
mpBMRI: Multiparametric bone MRI
NFR: Nuclear Fast Red
PSMA: Prostate specific membrane antigen
rFF: Relative fat fraction
VIBE: Volume interpolated breath-hold examination
WO: Water-only

## Declarations

### Guarantor

The scientific guarantor of this publication is Nicos Fotiadis.

### Statistics and biometry

No complex statistical methods were necessary for this paper.

### Methodology

• retrospective

• observational

• performed at one institution
